# Study on prevalence of ancylostomosis in dogs at Anand district, Gujarat, India

**DOI:** 10.14202/vetworld.2015.1405-1409

**Published:** 2015-12-17

**Authors:** Nilima N. Brahmbhatt, P. V. Patel, Jigar J. Hasnani, Suchit S. Pandya, B. P. Joshi

**Affiliations:** 1 Department of Veterinary Parasitology, College of Veterinary Science and Animal Husbandry, Anand Agricultural University, Anand, Gujarat, India; 2 Department of Veterinary Pathology, College of Veterinary Science and Animal Husbandry, Anand Agricultural University, Anand, Gujarat, India

**Keywords:** ancylostomosis, dog, fecal, prevalence rate, sedimentation technique

## Abstract

**Aim:**

This study was undertaken to derive the prevalence rate of ancylostomosis in dogs by a collection of fecal samples from Anand district.

**Materials and Methods:**

The fecal samples were collected from the dogs brought to the Hospital of Veterinary College (Teaching Veterinary Clinical Service Complex) and the surrounding areas of Anand district. On the day of collection, fecal samples were collected and brought to the Department of Veterinary Parasitology and processed for standard qualitative examination. The sedimentation technique was used to detect the presence of *Ancylostoma* spp. eggs in the samples.

**Result:**

The highest prevalence rate was observed in the month of May (36.66% fecal samples) and the lowest in the month of December (13.79% fecal samples) at Anand district.

**Conclusion:**

It can be concluded that heavy infection is present in Anand district especially in the season of summer followed by monsoon and the least in winter.

## Introduction

Dogs harbor a variety of intestinal parasites, some of which can also infect humans. In view of this, some of the dog parasites, such as *Toxocara canis* and *Ancylostoma* spp. are reported to be a significant public health problem, especially in developing countries and communities that are socioeconomically disadvantaged. In these communities, poor levels of hygiene and overcrowding, together with the lack of veterinary attention and zoonotic awareness, exacerbate the risk of disease transmission [1].

Ancylostomosis (hookworm disease) is a disease of worldwide distribution. The most widespread of all hookworm species is *Ancylostoma caninum* and it parasitizes dogs throughout the tropics and subtropics. Due to its high prevalence and its zoonotic significance, *A. caninum* has gained major importance in the field of veterinary as well as public health research. In recent years, the realization that *A. caninum* can cause human gut disease has sparked off renewed interest in its study [2].

Ancylostomosis occurs in warm and temperate climates, especially where there is adequate moisture. *A. caninum* and *Uncinaria stenocephala* infections are relatively common in pups, although the former is much more frequent [3]. *A. caninum* is the most pathogenic species of all hookworms in pet animals. The primary sign of hookworm infection and disease is anemia in dogs. *A. caninum* causes hemorrhagic diarrhea [4].

The stray and owned dogs play an important role in the transmission of these diseases although particular implication of each population is not clearly established. The transmission of these zoonotic helminths could be through direct contact or through indirect contact via infected food and water [5].

Our main objectives of this study are to derive month wise, season wise, age wise, breed wise, sex wise, and the overall prevalence of this worm so that prophylactic measures can be taken in future for protecting dogs from hookworm infection.

## Materials and Methods

### Ethical approval

Approval was given by the Research Advisory Committee and samples were collected as per standard sample collection procedure without harming or giving stress to any animals.

### Study area and sample collection

The study was carried out to ascertain the prevalence of ancylostomosis in dogs at Anand district of Gujarat. The study was undertaken for the period of 12-month from March-2014 to February-2015. A total of 392 fecal samples were collected from Anand district. Samples were collected from dogs brought to the Hospital of Veterinary College, Teaching Veterinary Clinical Service Complex (TVCC) and the surrounding areas of Anand district. The month wise, season wise, age wise, breed wise, sex wise, and overall prevalence were studied during the period. During whole of the study, the samples were collected during morning hours from the hospital of Veterinary College (TVCC) of Anand district, and samples were collected in small and clean sterilized polythene bags. The bags were numbered, ligated with rubber bands and were brought to the laboratory for further processing and examined for the presence of parasitic infection. For recording/findings of prevalence, fecal samples of dogs were collected for the detection and identification of eggs of *Ancylostoma* spp. as per standard method.

### Processing of fecal samples

Fecal samples were processed by qualitative examination *viz*; sedimentation technique for the identification of the egg in the laboratory [6].

### Statistical analysis

Chi square (χ^2^) test was used for analysis of prevalence data. For applying χ^2^ test, IBM SPSS 20.0 software was used.

### Meteorological data

Data were collected from Department of Agricultural Meteorology, BACA, AAU, Anand during the period of March-2014 to February-2015. Humidity and temperature have a direct relation to the growth of the parasites. In summer season, comparative higher humidity (70%) documented with 39°C temperature. In monsoon, 92% humidity with 34°C and in winter 90% humidity with 30°C temperature were notices which were also higher compared with previous years.

## Results

**Figure-1:**
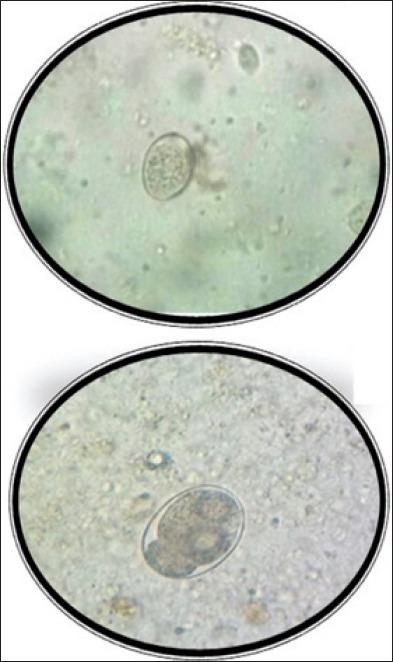
Eggs of *Ancylostoma caninum* in smear of fecal sample (×10) (×40).

Eggs were identified as of *Ancylostoma* spp. by sedimentation technique under the microscope in the laboratory on basis of its morphological characteristics (Figure-1). A total of 392 fecal samples were collected, out of which 90 (22.95%) fecal samples were found positive for the *Ancylostoma* spp. infection from Anand district.

### Month wise prevalence

**Figure-2:**
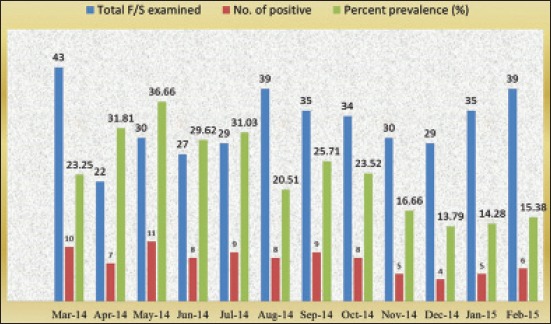
Month-wise prevalence of *Ancylostomosis* in dogs of Anand district by fecal examination.

The highest prevalence rate was observed in the month of May 36.66% and the lowest in the month of December 13.79% for the fecal samples (Figure-2) at Anand district. Hence, from March onward the intensity of the infection is increased up to the month of October and from November onward intensity of the infection is decreased up to the month of February.

### Season wise prevalence

In Anand district, seasonal prevalence found to be 29.50%, 24.81%, and 15.03% of summer, monsoon, and winter, respectively (Table-1) by fecal sample collection.

**Table-1:** Overall prevalence of *Ancylostomosis* in dogs of Anand district by fecal examination.

Parameters	Total F/S examined	No. of positive	Percent prevalence
Season			
Summer	122	36	29.50
Monsoon	137	34	24.81
Winter	133	20	15.03
Total	392	90	22.95
(Chi square=5.000, df=2), p=0.082 (Non-significant) (p<0.05=Significant) (p>0.05=Non-significant)			
Age (year)			
Young age (<1)	161	58	36.02
Middle age (1-7)	130	21	16.15
Old age (>7)	101	11	10.89
Total	392	90	22.95
(Chi square=16.66, df=2), p=0.00 (Significant) (p<0.05=Significant) (p>0.05=Non-significant)			
Sex			
Male	221	65	29.41
Female	171	25	14.61
Total	392	90	22.95
(Chi square=4.455, df=1), p=0.035 (Significant) (p<0.05=Significant) (p>0.05=Non-significant)			
Breed			
German shepherd	77	19	24.67
Pomeranian	80	13	16.25
Doberman	78	11	14.10
Labrador retriever	87	17	19.54
Mongrel	70	30	42.85
Total	392	90	22.95
(Chi square=22.932), df=4, p=0.00 (Significant) (p<0.05=Significant) (p>0.05=Non-significant)			

### Age wise prevalence

The occurrence of *Ancylostoma* was more frequently recorded in dogs from young age (<1 year) followed by middle age (1-7 year) and lowest in old age (>7 year) by examining fecal samples of Anand district. The age wise prevalence of 36.02% in young age, 16.15% in middle age, and 10.89% in old age from the fecal samples (Table-1).

### Sex wise prevalence

Overall the highest prevalence of *Ancylostomosis* was noticed in male and lowest in female by examining fecal samples of Anand district. The sex wise prevalence of 29.41% in male and 14.61% in female from the fecal samples (Table-1).

### Breed wise prevalence

The highest prevalence of ancylostomosis was noticed in mongrel (stray dog) and lowest in a doberman by examining fecal samples of Anand district. The breed wise prevalence of 42.85% in mongrel followed by German Shepherd (24.67%), Labrador retriever (19.54%), Pomeranian (16.25%), and Doberman (14.10%) from the fecal samples (Table-1).

### Overall prevalence

The overall prevalence rate was found to be 22.95% (90) from the fecal samples of Anand district (Table-1).

## Discussion

Month-wise prevalence was recorded by Oliveira-Sequeira *et al*. [7] who reported peak egg count at the beginning of summer with a peak occurrence during April and May which correlates with the present study because the higher prevalence in these months may be due to relatively higher environmental temperature and more rainfall in these months. The higher temperature is an important factor in the release of larvae from the eggs. Moreover, besides other risk factors associated with the disease, rainfall also influences the prevalence of the parasites. The higher prevalence of the parasites during the high rainfall, this may be associated due to sanitary problems as during the heavy rains, the water is lodges that may cause the higher prevalence during these months. In the May-2014, due to the changes in climate and sudden rainfall in investigated area, probably the prevalence rate is found high.

Seasonal prevalence found to be highest in summer followed by monsoon and the least in winter in the present study. The above findings were in accordance with the findings of Andresiuk *et al*. [8] and Tarafder and Samad [9]. As there is optimum required temperature and humidity that favors the development of eggs of hookworm and subsequently development of third stage infective larvae. Such ambient requirement favors the bionomics of hookworm larvae.

The age wise prevalence was in agreement with the Lefkaditis *et al*. [10] who examined 952 fecal samples, 18 (1.89%) recorded to be positive for *A. caninum* and 11 were belonged to male and the 7 female dogs, the 12 were belonged to young and the 6 to adult dogs. Sowemimo and Asaolu [11] recorded the prevalence of *Ancylostoma* spp. was observed to be the highest in dogs of age Group 0-6 months. Das *et al*. [12] who reported the hookworm infections were common in the age group of 2 months to 6 years (26.48%) in pet dogs.

The sex wise prevalence was recorded by Mitra *et al*. [13] and Oliveira-Sequeira *et al*. [7]. They have recorded higher infection in adult males than in adult females which support the findings of present study. Male has a high percentage (29.41%) of infection compare to female (14.61%). As hormones activity may play important role. This may also due to the individual hormonal status of male and female. It may require further investigation.

The breed wise highest prevalence of Ancylostomosis was noticed in mongrel (stray dog) and lowest in doberman by examining fecal samples. Similar findings were recorded by some other authors *viz*., Ramírez *et al*. [14], Das *et al*. [12], and Mahdy *et al*. [15]. They reported the *Ancylostoma* spp. infection was very common in mongrel dogs/urban stray dogs. Rural stray dogs had the highest prevalence followed by urban stray dogs. This may due to fact that pet dogs are kept under good hygienic conditions and provided well-balanced nutrition compared to stray (mongrel/s) dogs. In addition, the pet owners are considered to be aware of using anthelmintic in dogs.

The overall prevalence rate was found to be 22.95% (90) out of 392 fecal samples collected from Anand district. Similar findings were noticed by Jani *et al*. [16] who reported 26.9%, Oliveira-Sequeira *et al*. [7] reported the prevalence 23.6% *Ancylostoma spp*. in stray dog by faecal examinations, Ramírez *et al*. [14] reported (24.5%) prevalence of *Ancylostoma spp*. in dogs. Agnihotri *et al*. [17] mentioned eggs of the hookworms were found predominantly (19.06%) in dogs of Himachal Pradesh. This much high prevalence noticed may be due to heavy hot and humid climatic effect which is a most favorable condition for the survival of the parasite. The present findings are also in line with the Sowemimo and Asaolu [11] who mentioned prevalence 17.9% for *Ancylostoma* spp. in dogs from Nigeria; Singh *et al*. [18] recorded prevalence of *Ancylostoma* spp. was 19.32% in Punjab; Qadir *et al*. [19] found *A. caninum* infection was predominant (17.84%) in Jabalpur; Gugsa *et al*. [20] reported the prevalence rate of 24.00% *Ancylostoma* spp. in dogs. Apart from all these studies Ali *et al*. [21] encountered very low 3.22 % prevalence of *A. caninum* in contaminated soil samples collected from slums of Lahore and Godara *et al*. [22] who reported 13.3% dogs positive for hookworm (*A. caninum*) eggs in Jaipur. This may occur due to less hot humid climate availability in the study area.

## Conclusion

This study was undertaken to derive the prevalence rate of ancylostomosis in dogs by collection of fecal samples from Anand district. The fecal samples were collected from the dogs brought to the hospital of veterinary college (TVCC) and the surrounding areas of Anand district. Present studies show that incidence of *A. caninum* is higher in the month of May and lowest in December. In the summer season, prevalence was found to be highest followed by monsoon and winter season. As there is optimum required temperature and humidity that favors the development of eggs of hookworm and subsequently development of the third stage infective larvae. Such ambient requirement favors the bionomics of hookworm larvae. Male has a higher incidence than female. According to breed Mongrel showed higher incidence and Doberman having the lowest. As per the age group, young ones having highest infection compared to old age.

## Authors’ Contributions

This study is the major component of the work toward the M. V. Sc thesis of the first author NNB, under the guidance of PVP and JJH. SSP helped in sample collection from various abattoirs and also helped in technical writing of article. BPJ helped in thoroughly revision of the manuscript. All authors have read and approved the final version of the manuscript.
